# Genome-Wide Identification and Characterization of *gh/prl/sl* Family in *Cynoglossus semilaevis*

**DOI:** 10.3390/ijms26041585

**Published:** 2025-02-13

**Authors:** Min Zhang, Yuhong Shi, Zhe Wang, Zhangfan Chen, Xihong Li, Wenteng Xu, Na Wang

**Affiliations:** 1 College of Fisheries and Life Science, Shanghai Ocean University, Shanghai 201306, China; 17860361038@163.com; 2 State Key Laboratory of Mariculture Bioreading and Sustainable Goods, Yellow Sea Fisheries Research Institute, Chinese Academy of Fishery Sciences, Qingdao 266071, China; shiyuhong@zjou.edu.cn (Y.S.); wz17664081160@163.com (Z.W.); chenzf@ysfri.ac.cn (Z.C.); lixh@ysfri.ac.cn (X.L.); xuwt@ysfri.ac.cn (W.X.); 3 Laboratory for Marine Fisheries Science and Food Production Processes, Qingdao Marine Science and Technology Center, Qingdao 266237, China

**Keywords:** *gh*/*prl*/*sl* family, growth hormone receptor, prolactin receptor, Chinese tongue sole (*Cynoglossus semilaevis*), pituitary

## Abstract

The Chinese tongue sole (*Cynoglossus semilaevis*) is a marine flatfish of significant economic value, characterized by pronounced female-biased sexual size dimorphism (SSD). Sexual differences of cell number and gene expression within the PIT-1 lineage of the pituitary gland may be crucial for interpreting the female-biased SSD of *C. semilaevis*. Among hormones secreted by PIT-1 cell lineage, growth hormone (*gh*), prolactin (*prl*), prolactin 2 (*prl2*), and somatolactin (*sl*) comprise a gene family within the extensive superfamily of class-1 helical cytokines. To better understand the function of the *gh/prl/sl* in teleost SSD, we firstly identified five genes of the *gh/prl/sl* family (*gh*, *sl*, *prl*, *prl2a*, and *prl2b*) and their receptors (*ghra*, *ghrb*, *prlra*, *prlrb*, and *prlr*-*like*) from *C. semilaevis* at the genome-wide level. Phylogenetic analyses revealed that the *gh*/*prl*/*sl* family and their receptors were each clustered into five distinct groups. More microsatellites were revealed in the intron 2 of *gh* gene of female rather than the male and pseudo-male individuals, which is positively correlated with its sexual expression pattern. Interaction network prediction indicated that *gh*, *prl*, and *sl* may collectively contribute to individual growth and development. A FRET experiment showed that *ghra* can act as a receptor for *sl*. Additionally, the transcripts of the *gh*/*prl/sl* family and their receptors exhibited varying abundances in the pituitary, brain, gonad, and liver of both female and male *C. semilaevis*, with most ligands showing the highest abundance in the female pituitary. Furthermore, *gh* and *sl* were found to be maternally expressed. The knock-down of *gh*, *prl*, and *sl* in the pituitary cells could lead to the expression change of *igf1*, *c-fos*, and *sos2*. This study provided a foundation for further functional characterization of the *gh*/*prl/sl* gene family, contributing to a deeper understanding of the growth and reproductive mechanisms in *C. semilaevis*.

## 1. Introduction

Sexual size dimorphism (SSD) has been widely observed in animals including mammals, birds, reptiles, and fishes, characterized by different body or segment sizes in different sexes [1,2,3]. For example, more than 600 fish species exhibit obvious SSD [4], and this phenomenon in farmed fish species could cause growth disadvantages within a single sex, then leading to a decrease in production. Thus, an increasing number of studies have focused on elucidating the molecular mechanism of SSD in farmed fish, including Japanese flounder (*Paralichthys olivaceus*) [5], Mandarin fish (*Siniperca chuatsi*) [6], and Chinese tongue sole (*Cynoglossus semilaevis*) [7,8].

Importantly, our recent integration analysis by scRNA-seq and scATAC-seq revealed that the differences in the cell number and gene expression pattern of the pituitary gland are essential for interpreting female-biased SSD in *C. semilaevis*, a female heterogamete flatfish. Specifically, pituitary-specific POU homeodomain transcription factor 1 (PIT-1) cell lineages capture our interest because the mutation of PIT-1 in mammals could result in the failure of PIT-1 lineage differentiation, subsequently leading to a dwarf phenotype [9]. In contrast to three kinds of PIT-1 sublineages in mammals somatotrophs, lactotrophs, and thyrotropes, the fourth cell type, somatolactotrophs, were exclusively found in teleost [10,11], including *C. semilaevis*. Correspondingly, somatotrophs, lactotrophs, somatolactotrophs, and thyrotropes separately secrete growth hormone (*gh*), prolactin (*prl*), somatolactin (*sl*), and thyrotropic hormone (*tsh*), among which, *gh*, *prl*, and *sl* belong to the same hormone family and are involved in multiple effects including growth regulation, metabolism, energy balance, and osmoregulation [12].

The *gh*/*prl*/sl family presumably evolved from a common ancestor through gene duplication [13]. They share similarities in gene and protein tertiary structures, featuring four helices. *Gh* was first isolated from the human pituitary gland, and its mutation led to a disorder characterized by short stature [14]. Increasing evidence has indicated its multiple roles in regulating growth, metabolism, and reproduction of vertebrates including teleost [15,16]. The heterogeneity of *gh* has been identified in humans at the genome, mRNA, and post-transcriptional modification levels [17]. Similarly, two duplicated isoforms of *gh* genes are discovered in fish species, including salmonids and tilapias [18].

*Prl* was originally described as a polypeptide hormone to promote lactation [19]. Later studies found that *prl* was also involved in luteal function, reproductive behavior, immune response, osmoregulation, and angiogenesis [20,21]. In teleost, *prl* was first isolated from chum salmon (*Oncorhynchus keta*) and has a typical gene structure similar to mammals [22]. So far, three duplicated isoforms of *prl* genes have been discovered in teleost: *prl*, *prl2a*, and *prl2b* [23].

*Sl* was first found in the pituitary gland of Atlantic cod (*Gadus morhua*) and Japanese flounder (*Paralichthys olivaceus*) [24]. Two isoforms have been isolated, with *slα* being present in all fish species, and *slβ* being found only in a limited number of fish species [25]. Like *gh* and *prl*, *sl* have multiple functions in the gonad development, spawning, and body color formation [26,27].

Commonly, *gh*/*prl*/*sl* genes exert multiple biological effects by binding to single transmembrane domain receptors. Binding subsequently causes receptor dimerization and activates Janus kinase 2 (JAK2), a tyrosine kinase that initiates JAK-STAT signaling [28]. Different from two isoforms of *ghr* transcripts in humans [29], in teleost, *ghr* is divided into two branches: *ghr1* (type I *GHR*) and *ghr2* (type II *GHR*) [30]; two kinds of *ghr* genes have duplicated in teleost including salmonids, fugu, and zebrafish [31]. Similarly, two duplicated *prlr* genes (*prlra* and *prlrb*) were also discovered in Nile tilapia (*Oreochromis niloticus*) [32], goldfish (*Carassius auratus*) [33], sea bream (*Sparus auratus*) [34], and rainbow trout (*Oncorhynchus mykiss*) [35]. *Slr* has been reported in masu salmon (*Oncorhynchus masou*), medaka, and other fish species [36], with only one gene.

Given their high structural conservation, the one-to-one correspondence between *gh*/*prl*/*sl* ligands and receptors cannot be easily derived. To date, there is still much controversy regarding their binding relationship. For instance, in zebrafish, it was found that two *sl* genes did not physically interact with *ghr1*, while *slr* and *ghr* could both interact with *gh* of medaka [37].

To better understand the function of the *gh*/*prl*/*sl* family in teleost SSD, we first identified the members of the *gh*/*prl*/*sl* family and their receptors at the genome-wide level. Subsequently, a phylogenetic tree was constructed to reveal evolutionary relationships. In addition, conserved domains/motifs and protein interaction networks were analyzed. As well as their spatiotemporal expression patterns were detected, and the interaction of ligand receptors within the *gh*/*prl*/*sl* family was explored. Finally, the knock-down effect of important ligands on the downstream genes was studied.

## 2. Results

### 2.1. Identification and Sequence Characterization of gh/prl/sl Ligand and Receptor Family Members

A domain search of the whole genome of *C. semilaevis* identified five genes in the *gh*/*prl*/*sl* family: *gh*, *sl*, *prl*, *prl2b*, and *prl2a* (Table 1), located in different autosomes. In addition, the ORF sequences of these genes ranged in length from 603 bp to 762 bp, encoding 200–253 amino acids. The predicted MWs and pIs were 23.15–28.72 kDa and 5.76–8.32, respectively.

Similarly, five *gh*/*prl*/*sl* receptors including *ghra*, *ghrb*, *prlra, prlrb*, and *prlr-like* were identified from *C. semilaevis*. *Ghra* and *prlrb* are located on the Z chromosome. The ORF sequences of these genes ranged in length from 1334 bp to 1902 bp, encoding 443–663 amino acids. The predicted MWs and pIs were 62.41–70.712 kDa and 4.69–5.34, respectively.

### 2.2. Phylogenetic Analysis of gh/prl/sl Family

**Figure 1:**
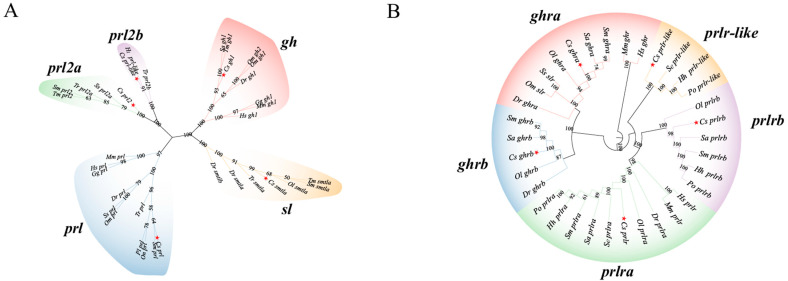
Phylogenetic analysis for *gh*/*prl*/*sl* family members and their receptors. (**A**) Phylogenetic tree of *gh*/*prl/sl* family member from *Cynoglossus semilaevis* (*Cs*), *Homo sapiens* (*Hs*), *Danio rerio* (*Dr*), *Mus musculus* (*Mm*), *Gallus gallus* (*Gg*), *Oncorhynchus mykiss* (*Om*), *Oreochromis niloticus* (*On*), *Sparus aurate* (*Sa*), *Poecilia latipinna* (*Pl*), *Takifugu rubripes* (Tr), *Thunnus maccoyii* (*Tm*), *Salmao salar* (*Ss*), *Hippoglossus stenolepis* (*Ht*), *Scophthalmus maximus (Sm),* and *Oryzias latipes (Ol)*. A phylogenetic tree with a bootstrap value of 1000 was constructed using MEGA 11.0, using the neighbor-joining method. The 5 sub-clusters are represented in different colors. The *gh*, *prl* and *sl* of *C. semilaevis* are marked with red stars. (**B**) Phylogenetic tree of *gh*/*prl*/*sl* receptor family member from *Cynoglossus semilaevis* (*Cs*), *Homo sapiens* (*Hs*), *Danio rerio* (*Dr*), *Mus musculus* (*Mm*), *Hippoglossus hippoglossus* (*Hh*), *Sole senegalensis* (*Se*), *Paralichthys olivaceus* (*Po*), *Gallus gallus* (*Gg*), *Oncorhynchus mykiss* (*Om*), *Sparus aurate* (*Sa*), *Scophthalmus maximus (Sm*), and *Oryzias latipe (Ol*). The *ghra*, *ghrb*, *prlra*, *prlrb*, *prlr*-*like* of *C. semilaevis* are marked red stars. The five sub-clusters are represented in different colors.

To investigate the evolutionary relationships of *gh*/*prl*/*sl* family members, a phylogenetic tree was constructed with homology proteins retrieved from the NCBI database (Figure 1A). The genetic relationship between *sl* and *gh* is relatively close. Interestingly, *sl* sequence was only found in fish, but not in *Homo sapiens*, *Mus musculus*, and *Gallus gallus*. As shown in Figure 1B, no receptor for *sl* was found in *C. semilaevis*. However, *slr* was found in *Oncorhynchus masou* and *Salmo salar* and clustered with *ghr*. This shows that the relationship between *slr* and *ghr* is close and conservative. We found that there are three receptors for *prl* in *Cynoglossus semilaevis*, *Hippoglossus hippoglossus*, *Sole senegalensis*, and *Paralichthys olivaceus*. However, only *prlra* and *prlrb* were found in zebrafish and *Sparus aurata*.

### 2.3. Conserved Domain, Gene Structure and Motif Composition

**Figure 2:**
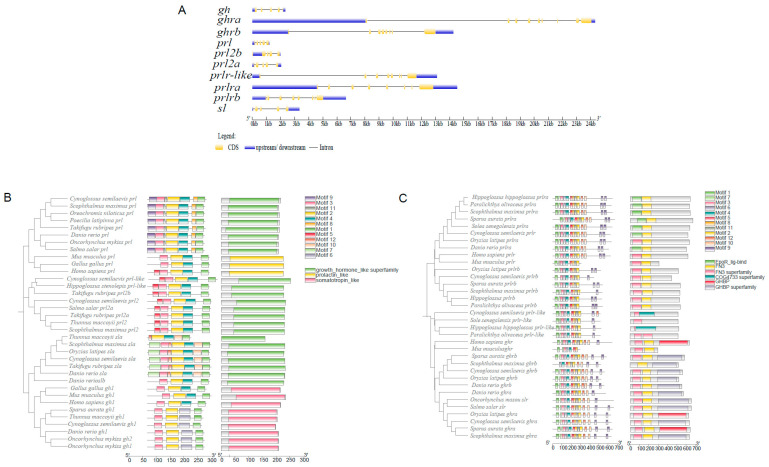
Gene structure and conserved domain analysis of growth hormone/prolactin ligand and receptor family members. (**A**) Gene structure. The yellow and blue rectangles represent the exons and UTR regions, respectively, the gray line represents the introns. (**B**) Conserved domain of *gh*/*prl*/*sl* ligand family. Different colored boxes represent the conserved domains and motifs. (**C**) Conserved domain of *gh*/*prl*/*sl* receptor family. Different colored boxes represent the conserved domains and motifs.

To further understand the structural diversity of *gh*, *sl*, and *prl* genes in *C. semilaevis*, the exon–intron of each gene was characterized. These genes possessed 5–6 exons and 4–5 introns (Figure 2A). As shown in Figure 2B, *gh*, *sl*, and *prl* in mammals and fish all contain a domain that belongs to the growth hormone superfamily. A total of 12 conserved motifs were searched from *gh*/*prl*/*sl* family, which showed that the sequences were relatively conserved. The length of these motifs ranged from 200 to 253 amino acids, as shown in Figure 2B. Three conserved motifs (Motif 3, Motif 2, and Motif 1) existed in almost all members, with the order of Motif 3, Motif 2, and Motif 1. This indicates that the *gh*/*prl*/*sl* family has highly conserved sequences in the process of evolution. In addition, the *gh*, *sl*, and *prl2* of *C. semilaevis* owned their unique motifs, indicating that there may be functional differences among different sub-clusters (Figure 2B).

The receptors all have nine exons and eight introns, suggesting high gene structural conservation throughout the evolutionary process. The *ghra* and *ghrb* all have FN3 and GHBP domain. In addition, the *prlr* of tongue sole have EpoR_lig-bind domain and FN3 domain. Six conserved motifs (Motif 6, Motif 4, Motif 5, Motif 8, and Motif 11) existed in almost all members, and they were all arranged in the order of Motif 6, Motif 4, Motif 5, Motif 8, and Motif 11, as shown in Figure 2C.

### 2.4. Protein–Protein Interaction (PPI) Network Analysis and Tissue Distribution of gh/prl/sl Family Members in the C. semilaevis

**Figure 3:**
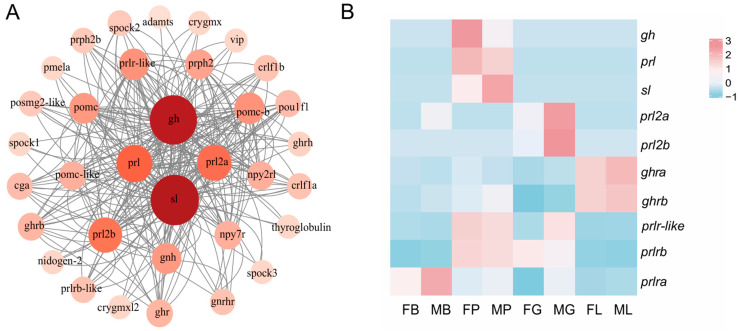
Protein–protein interaction (PPI) network analysis and tissue distribution of *gh*/*prl*/*sl* family and their receptors in the *C. semilaevis*. (**A**). Interaction network of *gh*/*prl*/*sl* family member. Nodes indicated the interactive proteins; lines indicated both functional and physical protein associations. Different colors of nodes reflect the number of interactions, with intense red indicating more interactions than pale red. (**B**). Heatmap of *gh*/*prl/sl* family members and their receptors, mRNA abundances in different tissues of healthy female and male Chinese tongue sole. FP: female pituitary; MP: male pituitary; FB: female brain; MB: male brain; FG: female gonad; MG: male gonad; FL: female liver; ML: male liver. The expression levels were quantified as FPKM based on RNA-Seq. Gene expression levels were color coded from low (blue) to high (red). Each row represented one gene (listed on the right).

To elucidate the biological activity and intricate regulatory network of the *gh*/*prl*/*sl* family, a protein–protein interaction (PPI) network was constructed comprising 40 nodes and 148 edges (Figure 3A). According to the PPI network, the *pou1f1* transcription factor was identified as a key regulator involved in the expression, and the main biological processes involved are response to growth hormone, growth hormone receptor signaling pathway, positive regulation of receptor signaling pathway via JAK-STAT, cellular response to peptide, and cytokine-mediated signaling pathway. Additionally, the primary KEGG pathways associated with these processes are cytokine–cytokine receptor interaction and neuroactive ligand–receptor interaction. For example, the binding of *gh* and *ghr*, as well as the binding of *prl* and *prlr*, all involve JAK-STAT, cytokine–cytokine receptor interaction, and neuroactive ligand–receptor interaction. These processes and pathways are interconnected and play critical roles in various physiological and pathological conditions.

To better understand the potential roles of *gh*, *prl*, *sl*, and their receptors, their expression patterns were illustrated by using of 2-year-old female and male *C. semilaevis* RNA-seq data [8] (Figure 3B). The transcripts of three ligands (*gh*, *prl* and *sl*) showed different mRNA abundances in the pituitary of female and male *C. semilaevis*. The *prl2a* and *prl2a* were found in the gonad with the highest abundance. As for receptors, *ghrb*, and *prlr*-*like* are primarily expressed in the liver, while *prlrb* and *prlra* mainly expressed in the pituitary and brain, respectively.

### 2.5. Spatiotemporal Expression Patterns of gh, prl, and sl

**Figure 4:**
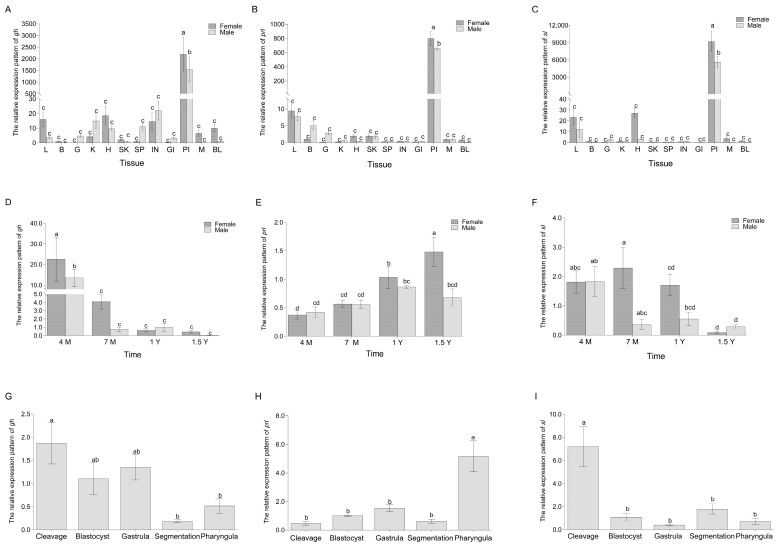
The spatiotemporal expression patterns of *gh*, *prl*, and *sl* transcripts. (**A**–**C**) Relative expression detected by qPCR of *gh*, *prl* and *sl* mRNA, respectively, in liver (L), brain (B), gonad (G), kidney (K), heart (H), skin (SK), spleen (SP), intestine (IN), gills (GI), pituitary (PI), muscle (M), and blood (BL) of different genders. Values with different letters differ significantly (*p* < 0.05) and using one-way ANOVA and multiple comparison by Wohler and Duncan methods. (**D**–**F**) represented the relative expression patterns of *gh* and *prl*, *sl* mRNA in 4 Months, 7 Months, 1 Year, 1.5 Years. The dark-gray and gray separately represented female, male *C. semilaevis*. (**G**–**I**) showed the relative expression levels of *gh*, *prl*, and *sl* genes in embryonic development stages which is cleavage period, blastocyst period, gastrula period, segmentation period, and pharyngula period. The data were analyzed with SPSS 25.0 (IBM Corp., Armonk, NY, USA) using one-way ANOVA and multiple comparisons by Wohler and Duncan methods, and *p*-value < 0.05 was considered the threshold for statistical significance.

To further understand the detailed expression pattern of three pituitary abundant ligands, the expression characteristics of *gh*, *prl*, and *sl* in twelve tissues of male and female *C. semilaevis* were detected by qPCR (Figure 4A–C). Specifically, the expressions of *gh*, *prl*, and *sl* in the pituitary were significantly higher (*p* = 0.001, *p* = 0.001, *p* = 0.001) than in other tissues. On the other hand, *gh*, *prl*, and *sl* exhibited higher expression levels in the pituitary of 1-year-old female fish than in the pituitary of male fish.

The expression characteristics of *gh*, *prl*, and *sl* in four development stages (4 Months, 7 Months, 1 Year, 1.5 Years) of male and female *C. semilaevis* were further detected (Figure 4D–F). Specifically, the expressions of *gh* in 4 months were significantly higher (*p* = 0.002) than in other times. On the other hand, *sl* and *prl* exhibit high expression levels at the ages of 7 months and 1.5 years.

Furthermore, *gh* and *sl* are highly expressed during the cleavage stage. *Prl* was predominantly observed during the pharyngula period, indicating that *gh* and *sl* exhibited maternal expression (Figure 4G–I).

### 2.6. Differences in Genomic Structure Between Male, Female, and Pseudo-Male gh

**Figure 5:**
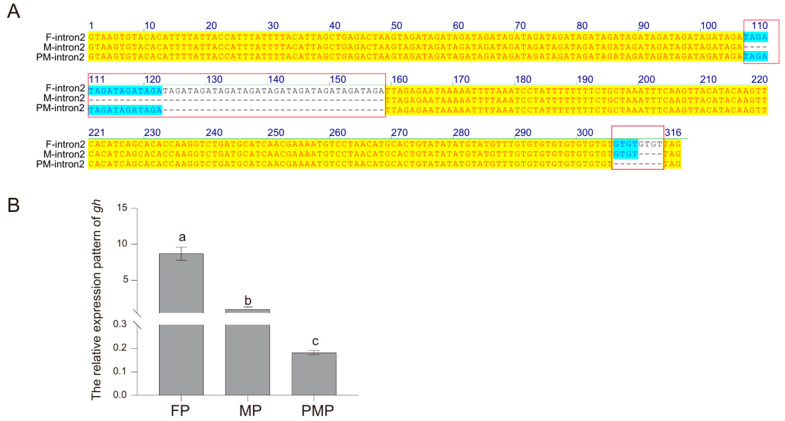
Comparison diagram of the *gh* genomes of males, females, and pseudo-males. (**A**) It is the multiple sequence alignment diagram, with yellow shading indicating a 100% similarity, blue indicating a similarity greater than 50%, and microsatellite differences highlighted in red boxes. (**B**) It represents the expression of *gh* in the pituitary glands of females (FP), males (MP), and pseudo-males (PMP) of 2 years old. Significant differences (*p* < 0.05) are marked by different lowercase letters (a,b,c), based on one-way ANOVA followed by Tukey’s post hoc test.

The *gh* genomic sequences obtained from the female, male, and pseudo-male *C. semilaevis* were 2336 bp, 2277, and 2278 bp, respectively. There was no significant difference in the genetic organization of the *gh* genes between the sexes, with six exons and five introns. However, the sexual differences were mainly within the second intron size produced by two kinds of microsatellites, “TAGA” and “GT” (Figure 5A). The number of “TAGA” in female, male and pseudo-male is 27, 14, and 18, respectively. In addition, there are twelve “GT” in females, ten in males, and eight in pseudo-males. Therefore, the size of the second introns in female, male, and pseudo-male fish were 316, 260, and 272 bp, respectively. The qPCR experiment further showed that *gh* had the highest expression level in female fish and the lowest expression level in pseudo-male fish (Figure 5B).

### 2.7. The Fluorescence Resonance Energy Transfer (FRET) Efficiency for the Binding Relationship Between gh, sl and ghra, ghrb

**Figure A1:**
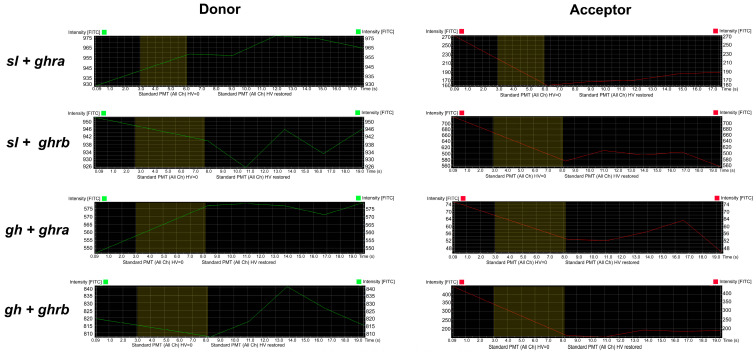
The fluorescence intensity change curve before and after the receptor–ligand interaction is quenched by laser. Green lines represent donor and red lines represent acceptor. The yellow area indicates the start of laser quenching, and after yellow is the end of quenching.

To validate the binding relationship between ligands and receptors, FRET was employed according to previous study [38,39]. Briefly, we obtained the fluorescence intensity change curve and corresponding values using the laser confocal microscope’s fret AB module. Based on the results in Table 2, the FRET efficiency of *sl* and *ghra* co-transfection is comparable to that of *gh* and *ghra* co-transfection, and notably higher than that of *sl* and *ghrb* co-transfection. This suggests that *sl* and *ghra* interact with each other. When comparing the FRET effectiveness of *gh* and *ghrb*, *gh* and *ghra* have a greater efficiency, implying that the binding of *gh* and *ghra* is stronger than that of *ghrb* in *C. semilaevis*. The change curve of donor acceptor fluorescence intensity is shown in Figure A1.

### 2.8. Knock-Down Effects on gh, sl, prl, and Other Related Genes by RNAi Transfection in Pituitary Cells

**Figure 6:**
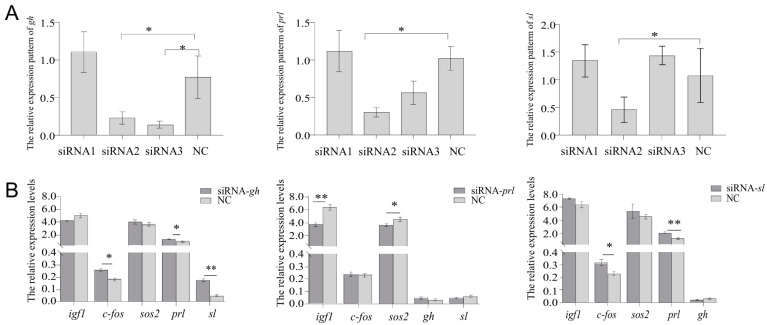
The knock-down effect of *gh*, *prl*, and *sl* on the female C. *semilaevis* pituitary cells. (**A**) Interference efficiency of *gh*, *prl* and *sl* siRNA. (**B**) The expression patterns of genes in female pituitary cells after transfection with *gh*, *sl*, and *prl* siRNA. Dark gray represents the gene knockout site, and light gray represents the NC negative control. The data were analyzed with SPSS 25.0 (IBM Corp., Armonk, NY, USA) using *t*-test. The data of each downstream gene were compared with NC and *p*-value < 0.05 was considered the threshold for statistical significance and indicated by *. (**, *p* < 0.01).

Three siRNA sites were each designed on the CDS region of *gh*, *prl*, and *sl* genes of *Cynoglossus semilaevis* by Sangon Biotech (Shanghai, China) Co., Ltd., named *gh*-siRNA1, *gh*-siRNA2, *gh*-siRNA3, *prl*-siRNA1, *prl*-siRNA2, *prl*-siRNA2, *sl*-siRNA1, *sl*-siRNA2, and *sl*-siRNA3. Female C. *semilaevis* pituitary cells were used for RNAi experiments to investigate the knock-down impact of *gh*, *prl*, and *sl*. Figure 6A shows that the 3rd site of *gh*, the 2nd site of *prl*, and the 2nd site of *sl* all had strong knock-down effects, with inhibition efficiencies more than 50%. And genes associated with *prl*, *gh*, and *sl* were screened based on the prolactin signaling pathway and growth hormone receptor pathway. After the transfection of siRNA for ligands, the qPCR analysis was performed to evaluate the expression levels of *gh*, *prl*, *sl* and their related genes: insulin-like growth factor 1 (*igf1*), proto-oncogene c-Fos-like (*c-fos*), and son of sevenless homolog 2 (*sos2*). The NC is the negative control. The results showed that *c-fos*, *prl*, and *sl* increased after the knock-down of *gh*. When *prl* was interfered, the decrease in *igf1* and *sos2* were observed. After *sl* was knocked down, *prl* and *c-fos* increased to varying degrees (Figure 6B).

## 3. Discussion

Sexual dimorphism, characterized by morphological, physiological, and behavioral differences between males and females, is prevalent throughout the animal kingdom [40]. To interpret sexual size dimorphism (SSD) of *C. semilaevis*, transcriptomics and molecular experiments have identified numerous genes and pathways including steroid biosynthesis, cell cycle regulation, and hippo signaling pathways, which exhibit significantly different expression levels between the sexes [8,41,42]. Given the observation of differentially expressed *gh*/*prl*/*sl* family genes across sexes and the extensive biological functions of this gene family in fish growth, development, and reproduction [43,44], a comprehensive genomic identification of *gh*/*prl*/*sl* family genes from *C. semilaevis* was conducted to facilitate the investigation of these genes’ involvement in fish SSD.

From the phylogenetic analysis, five main clusters were identified—*gh, prl*, *prl2a*, *prl2b,* and *sl*—diverging early in vertebrate evolution, which is agreement with previous phylogeny research [45]. The origin of the *gh*/*prl*/*sl* family can be traced back to early branched vertebrates, with *gh* and *prl-like* members identified in jawless vertebrates (*agnathans*), such as the sea lamprey (*Petromyzon marinus*) [46]. These ancient members provide significant insights into the early evolution of the *gh*/*prl*/*sl* family. In teleost, an additional genome-wide duplication event (3R) also occurred, resulting in the replication of the *sl* gene and the emergence of two subtypes: *sl*α and *sl*β [13,47]. We only identified *sla* in *C. semilaevis*, but *sl*β was only reported in some teleost such as goldfish, zebrafish, and grass carp [26]. The identification of three *prl* genes and one *sl* gene in C. *semilaevis* demonstrated their derivation from the first two genome-wide duplication events (1R and 2R) [26].

Interestingly, the sexual sequence differences are primarily attributed to the microsatellite (TAGA) located in the second intron, which is similar with previous study in *C. semilaevis* [48]. However, the identification of microsatellite differences in the pseudo-male is reported for the first time. In humans, GT microsatellites within the promoter of *ghr* gene could exert both cis- and trans- effects on *ghr* in a sex-specific manner [49]. In present study, the numbers of “TAGA” in female, male, and pseudo-male individuals are positively related with their expression levels, which implied that this intronic SSR could affect gene transcription [50], although the regulation mechanism needs further exploration.

The receptors of the *gh*/*prl*/*sl* hormone family also evolved from a common ancestral source, akin to ligands. In *C. semilaevis*, it has two different *ghr* genes (*ghr1* and *ghr2*). *Slr* is not found in *C. semilaevis,* and the first documentation of *sl* receptors originated from studies on salmon (*Salmo salar*), placing *sl* receptors within the evolutionary lineage of *ghra* [51]. To gain further insights on the interaction among *ghra*, *ghrb,* and *sl*, a fluorescence resonance energy transfer (FRET) experiment was employed, and the interaction between *sl* and *ghra* was slightly higher than that of *ghrb*, which implied that *sl* may bind to *ghra*. It is similar with the binding relationship in zebrafish [52].

Through PPI network analysis, we found that there are interactions between *gh, prl*, and *sl* and that there is not a one-to-one correspondence between receptors and ligands; for example, in humans, *gh* binds both *ghr* and *prlr* [53]. In the context of the PPI network, we focused on three genes—*pou1f1*, vasoactive intestinal peptide (*vip*), and Pro-opiomelanocortin (*pomc*)—which are interconnected with the *gh*/*prl*/*sl* family. *Pou1f1* is involved in pituitary development, body growth, and the production of several hormone genes in fish. It can bind to the promoter region and stimulate the production of *gh, prl*, and *sl* [54]. As a growth and developmental regulator, *vip* plays a critical role as a neuronal survival factor [55] and stimulates *prl* release [56]. As a common precursor for adrenocorticotropic hormone (ACTH) and corticotropin-releasing hormone, *pomc* is also involved in the regulation of *gh* release [57]. In the future, the involvement of *pou1f1, vip,* and *pomc* in the function of *gh*/*prl*/*sl* family deserves further in-depth study.

The pituitary gland-biased distribution pattern of *gh, prl,* and *sl* were noticed in the present study. Specifically, *gh* exhibited a declining trajectory from 4 months until the age of 1.5 years, with female-biased expression, which is generally consistent with previous reports [48]. In zebrafish (*Danio rerio*) and turbot (*Scophthalmus maximus*), *prl* showed significant expression levels in the pituitary gland, while demonstrating minimal expression in brain tissue and negligible expression in other tissues [58,59]. Compared with *prl*, only few transcripts of *prl2a* and *prl2b* were found in the gonads, with male-biased expression. It is inferred that the action of prolactin is mainly through *prl*. In fish, *prl* has been shown to stimulate steroidogenesis in both males and females, such as tilapia (*Oreochromis mossambicus*) [60] and chum salmon (*Oncorhynchus keta*) [61].

The detection of *gh* and *sl* in the cleavage stage suggested the involvement of these two genes in the early embryonic development process. Similarly, the maternal mRNA of *gh* and *prl* was also detected in rainbow trout (*Oncorhynchus mykiss)* and zebrafish [62,63]. The growth hormone/prolactin family may have a collaborative role in early embryonic development; however, the precise mechanisms by which they contribute to this process necessitate additional investigation.

It was found that after *gh* knocking down, *c-fos*, *prl* and *sl* were activated. The *gh* gene can regulate the transcription of *c-fos* [64], and *c-fos* deficiency perturbs normal development of bone, cartilage, and the hematopoietic system [65]. The increase of *prl* and *sl* implied their compensatory effect to *gh*. We discovered that after knocking out *sl*, *c-fos* and *prl* up-regulated, suggesting that *prl* and *sl* shared similar functions. After *prl* was knocked down, the down-regulation of *igf1* and *sos2* were detected. *Prl* regulates mammary epithelial cell survival and death via influencing the production of *igf-1* and its binding protein *igfbp-5* [66]. *Prl* and *igf-1* regulate immune responses through synergistic action [67]. Further exploration of complex interaction among these ligands and other growth-related genes would be helpful to understand sexual size dimorphism in fish species.

## 4. Materials and Methods

### 4.1. Animal Euthanasia and Ethics Statement

In this study, the fish were anesthetized with MS-222 to alleviate pain. All experimental procedures were performed in accordance with the Institutional Animal Care and Use Committee of the Yellow Sea Fisheries Research Institute.

### 4.2. Fish Preparation and Sample Collection

Fish samples were obtained from the Haiyang Yellow Sea Fisheries Co., Ltd., Qingdao, China. After dissection, tissues including gonads, liver, spleen, pituitary, brain, muscle, gill, intestine, heart, skin, and kidney were collected from three female and three male individuals of 4-month-old, 7-month-old, 1-year-old, and 1.5-year-old of *C. semilaevis* individuals. All tissues were stored in an RNA preservation solution (TaKaRa, Osaka, Japan), and total RNA was extracted using TRIzol reagent (Invitrogen, Carlsbad, CA, USA). The integrity, concentration, and quality of the isolated RNA were determined by agarose gel electrophoresis and NanoPlus (GE, Boston, MA, USA). The cDNA was synthesized using TB Green^®^Premix Ex Taq™ (TaKaRa, Osaka, Japan) and stored at −20 °C.

### 4.3. Sequence Retrieval and Analyses

The Hormone_1 domain (PF00103) and GHBP(PF12772) were downloaded from the PFAM database (http://pfam.xfam.org/, accessed on 6 October 2023) and used to identify the growth hormone/prolactin ligand and receptor family members from the *C. semilaevis* genome. The available sequences of *C. semilaevis* and other species, including humans (*Homo sapiens*), zebrafish (*Danio rerio*), medaka (*Oryzias latipes*), mouse (*Mus musculus*), chicken (*Gallus gallus*), rainbow trout (*Oncorhynchus mykiss*), sea bream (*Sparus aurata*), Tiger Puffer (*Takifugu rubripes*), southern bluefin tuna (*Thunnus maccoyii*), and turbot (*Scophthalmus maximus*) were retrieved and confirmed in the National Center for Biotechnology Information (NCBI) database (https://www.ncbi.nlm.nih.gov/, accessed on 6 October 2023). The accession numbers for proteins have been presented in Appendix A Table A1. The molecular weights (MWs) and theoretical isoelectric points (pIs) were predicted using ExPASy (http://web.expasy.org/protparam/, accessed on 20 October 2023). The conserved domains were characterized using the PFAM database. Chromosomal locations and exon/intron gene structures were obtained from the NCBI database.

### 4.4. Phylogenetic Tree and Structural Analysis

Multiple sequence alignments of the identified growth hormone/prolactin ligand and receptor family member amino acid sequences from different species were performed using ClustalW version 2.0. A phylogenetic tree with a bootstrap value of 1000 was constructed using MEGA 11.0, using the neighbor-joining method. Using the Newick file retrieved from MEGA 11.0, the tree was further beautified and visualized in Chiplot (https://www.chiplot.online, accessed on 10 April 2024). Gene structure display server 2.0 (GSDS2.0, http://gsds.gao-lab.org/index.php, accessed on 20 April 2024) was used to analyze the exon/intron gene structure of *C. semilaevis* sequences. The conserved domains and motifs were, respectively, characterized by the Conserved Domain Database from the NCBI (https://www.ncbi.nlm.nih.gov/cdd, accessed on 6 May 2024) and MEME (Version 5.4.1, https://meme-suite.org/meme/tools/meme, accessed on 6 May 2024) programs, followed by visualization using the TB-tools 2.154.

### 4.5. Interaction Network Analysis and Tissue Expression Analysis

The protein–protein interaction (PPI) network was predicted based on the homology of *C. semilaevis* using the Search Tool for the Retrieval of Interacting Genes/Proteins (STRING) database (https://string-db.org/cgi, accessed on 1 October 2024) with a medium level of confidence (0.40). Cytoscape 3.10 software was used to visualize and exhibit the interaction network.

We utilized RNA-seq data of the brain, liver, gonads, and pituitary from 2-year-old fish [8] to understand the mRNA distribution in *C. semilaevis*. The relative mRNA abundances of the above-mentioned growth hormone/prolactin ligand and receptor family member genes were obtained from these data, and a heatmap was created using the Omicshare Tool (https://www.omicshare.com/tools, accessed on 12 October 2024) to visualize the expression profiles.

### 4.6. Spatiotemporal Expression Analysis

The qPCR primers (Table 3) were designed to detect the expression patterns of *gh, prl*, and *sl* in different tissues and at different times. In brief, 12 tissues, including the gonad, liver, spleen, brain, pituitary, muscle, gill, intestine, heart, skin, and kidney, were isolated from three female and three male 1-year-old *C. semilaevis*. We also examined gene expression during embryonic development. We collected samples from five stages of *C. semilaevis*, including the cleavage period, blastocyst period, gastrula period, segmentation period, and pharyngula period.

β-actin was selected as the internal reference gene. Amplification was accomplished using THUNDERBIRD™ Next SYBR^®^ qPCR Mix (Toyobo, Tokyo, Japan) on a 7500 Fast Real-Time PCR system (ABI, Los Angeles, CA, USA). The conditions were 95 °C for 30 s, 40 cycles of 95 °C for 5 s, and 60 °C for 34 s. Melting curve analysis confirmed the specificity of this reaction. The relative expression of each gene was analyzed using the 2^−ΔΔCt^ method. Statistical analyses were performed using the GraphPad Prismversion 10.1.2.

### 4.7. Genomic DNA Sequences for gh Gene from Female, Male, and Pseudo-Male C. semilaevis

The genomic DNA was extracted using the TIANamp Marine Animals DNA Kit (Tiangen, China) according to the manufacturer’s protocol. The integrity, concentration, and quality of the DNA were assessed using agarose gel electrophoresis and a Nano Vue Plus spectrophotometer (GE, USA). Primers, *gh*-DNA-F and *gh*-DNA-R (Table 3) were designed to amplify genomic DNA from three female, three male, and three pseudo-male fish. The amplification was performed using the KOD DNA polymerase enzyme (Toyobo, Tokyo, Japan) under the following thermal cycling conditions: an initial denaturation step at 94 °C for 2 min, 40 cycles of 98 °C for 10 s, 56 °C for 10 s, and 68 °C for 30 s. The PCR products were purified, cloned into the pEASY-T1 vector, and sequenced by Qingdao Ruibo Company, Qingdao, China. To explore the expression of *gh* gene in the pituitary of three genders, pituitary tissues from three biological replicates per group (two-year-old females, males, and pseudomales) were collected for quantitative real-time PCR (qPCR) analysis.

### 4.8. The Fluorescence Resonance Energy Transfer (FRET) Efficiency for the Binding Relationship Between sl and ghra

The coding sequences (CDS) of *sl*, *gh*, *ghra*, and *ghrb* were initially amplified using specific primers (*sl*-cds-F, *sl*-cds-R, *gh*-cds-F, *gh*-cds-R, *ghra*-cds-F, *ghra*-cds-R, *ghrb*-cds-F, *ghrb*-cds-R) as listed in Table 3. These amplified fragments were subcloned into pcDNA3.1-mNeonGreen and pcDNA3.1-mScarlet-I plasmids using HindIII restriction enzymes and the TOROIVD^®^ One-Step Fusion Cloning MIX Seamless Cloning Kit (TOROIVD, Shanghai, China). This process generated recombinant plasmids: pcDNA3.1-N-*sl*, pcDNA3.1-N-*gh*, pcDNA3.1-S-*ghra*, and pcDNA3.1-S-*ghrb*. HEK 293T cells were co-transfected with various plasmid combinations—pcDNA3.1-N-*sl*/pcDNA3.1-S-*ghra*, pcDNA3.1-N-*sl*/pcDNA3.1-S-*ghrb*, pcDNA3.1-N-*gh*/pcDNA3.1-S-*ghra*, and pcDNA3.1-N-*gh*/pcDNA3.1-S-*ghrb*—at a 1:2 ratio, with 2.5 μg of plasmid per well in 6-well plates, using the Lipo8000™ transfection reagent (Beyotime, Shanghai, China). After 48 h of transfection, fluorescence signals were visualized using a Nikon A1R HD25 laser confocal microscope (Nikon, Tokyo, Japan). The FRET (Förster Resonance Energy Transfer) Acceptor Bleaching method was employed to detect changes in donor fluorescence intensity. The FRET efficiency was calculated using the formula FRETeff = (D_post_ − D_pre_)/D_post_, where D_post_ represents the fluorescence intensity of the donor after quenching, and D_pre_ represents the intensity before quenching.

### 4.9. Design and Transfection of RNAi in Female C. semilaevis Pituitary Cell Lines

Based on the mRNA sequences of *gh*, *prl*, and *sl*, three specific small interfering RNA (siRNA) were designed and ordered from Sangon Biotech (Shanghai) Co., Ltd. (Sangon, Shanghai, China), as detailed in Table 3. The negative control (RNAi-NC) is provided by Sangon Biotech. Utilizing the RiboFECT™ CP Transfection Kit (Ribobio, Guangzhou, China), the negative control (RNAi-NC), positive control (RNAi-cy3), and the siRNA targeting were transfected into female pituitary cells. Specifically, 3 µL of siRNA (20 µM) was diluted in 60 µL of CP buffer and 5 µL of CP reagent, and the resulting mixture was added to each well of a 12-well plate. Forty-eight hours post-transfection, total RNA extraction, complementary DNA (cDNA) synthesis, and quantitative polymerase chain reaction (qPCR) were performed according to the previously described methods. The data were analyzed using SPSS version 25.0 (IBM Corp., Armonk, NY, USA), employing a *t*-test for statistical comparison. The expression levels of each downstream gene were compared to the negative control, with a *p*-value of less than 0.05 considered statistically significant.

## 5. Conclusions

In conclusion, we identified five *gh*/*prl*/*sl* family ligands and five receptor genes from Chinese tongue sole and characterized their evolutionary relationships. The genomic structure analysis for *gh* gene revealed that sexual differences mainly existed in number of intronic microsatellite “TAGA”, which might influence the expression pattern. The predominant expressions of *gh*, *sl*, and *prl* in the pituitary tissues were observed and their interaction with other factors including *pou1f1*, *vip*, and *pomc* warrants further research. The maternal expression of *gh* and *prl* implied that they had a collaborative role in early embryonic development. FRET experiments showed that *sl* and *ghra* may interact and exert their effects. Moreover, our research would be helpful to better understand the evolution and function of *gh/prl/sl* family and receptors in teleost.

## Figures and Tables

**Table 1:** Sequence features of growth hormone/prolactin ligand and receptor family members.

Name	Gene ID	Gene Length (bp)	ORF Length (bp)	Amino Length (aa)	MW (kDa)	pI	Chr	Location	No. of Exons
*gh*	103387754	2358	603	200	23.15	7.04	12	16,479,910–16,482,267	6
*sl*	103377636	3334	699	232	26.73	5.76	4	1,102,903–1,106,236	5
*prl*	103393669	1205	654	217	23.66	8.32	17	15,922,112–15,923,316	5
*prl2a*	103383000	2061	708	235	27.19	6.62	8	28,798,050–28,800,110	5
*prl2b*	103380354	1993	762	253	28.72	7.65	6	14,399,000–14,400,992	5
*ghra*	103397680	24,333	1902	633	70.71	4.69	Z	6,180,207–6,204,539	9
*ghrb*	103389092	14,280	1686	561	62.41	4.76	14	1,970,750–1,985,029	9
*prlra*	103390274	14,542	1860	619	69.58	5.34	14	25,242,433–25,256,974	9
*prlr-like*	103389689	13,111	1536	511	57.99	5.94	14	12,848,157–12,861,267	10
*prlrb*	103397545	6636	1334	443	49.03	6.64	Z	4,468,207–4,474,842	9

**Table 2:** The fluorescence resonance energy transfer efficiency for the binding relationship between *sl* and *ghra*, *ghrb*.

	*sl + ghra*	*sl + ghrb*	*gh + ghra*	*gh + ghrb*
Donor Pre	927	199	547.11	556
Donor Post	976.7	205	578.34	577
Acceptor Pre	272.6	721.04	160.74	817.76
Acceptor Post	159.6	573.48	57	455.22
Efficiency	0.050886	0.029268	0.0534	0.036

Note: The terms Donor post and Donor pre refer to the fluorescence intensity of the donor after and before quenching, respectively. *sl* + *ghra* represents the change in fluorescence intensity resulting from the co-transfection of pcDNA3.1-n-*sl* and pcDNA3.1-s-*ghra*. Similarly, *sl* + *ghrb* denotes the change in fluorescence intensity observed following the co-transfection of pcDNA3.1-n-*sl* and pcDNA3.1-s-*ghrb*. *gh* + *ghra* indicates the fluorescence intensity change before and after quenching for the co-transfection of pcDNA3.1-n-*gh* and pcDNA3.1-s-*ghra*. Lastly, *gh* + *ghrb* represents the fluorescence intensity change following the co-transfection of pcDNA3.1-n-*gh* and pcDNA3.1-s-*ghrb*. Efficiency is $FRETeff=\frac{Dpost-Dpre}{Dpost}$.

**Table 3:** The primers used in the present study.

Primer	Sequences (5′–3′)	Information
*gh*-F	ATCCACGCAGCCGGTTATAG	qPCR
*gh*-R	CTCATGCTTGTTGTCGGGGA	qPCR
*prl*-F	ATTCCAAGAGTCTGGGCGAC	qPCR
*prl* -R	CGATCTTGTGGGAATCCCGT	qPCR
*sl*-F	CTGCTTGTTTACCGTGAGCG	qPCR
*sl*-R	GGAGACGCAGTGGAAAAGGA	qPCR
*gh*-DNA-F	GTCAGAATCAGAACCAAACCA	DNA
*gh*-DNA-R	ACATATGCCGACAGAATGACA	DNA
βactin-F	TTCCAGCCTTCCTTCCTT	qPCR
βactin-R	TACCTCCAGACAGCACAG	qPCR
*gh*-cds-F	TAGCGTTTAAACTTAAGCTTATGGACAAACTTGTTTTACTGT	FRET
*gh*-cds-R	GAACCGTTGCCAGAGGATCCGGCGGTGCTTCCCTACAGGGTACAGTTAGCTTCT	FRET
*sl*-cds-F	TAGCGTTTAAACTTAAGCTTATGCATGCAATGATGACAGTA	FRET
*sl*-cds-R	GAACCGTTGCCAGAGGATCCGGCGGTGCTTCCTTATGCACAGTTGTACTTGTC	FRET
*ghra*-cds-F	TAGCGTTTAAACTTAAGCTTATGGCTATCCACTCACTCTC	FRET
*ghra*-cds-R	GAACCGTTGCCAGAGGATCCGGCGGTGCTTCCGCAAATTTCATGGTGAGAG	FRET
*ghrb*-cds-F	TAGCGTTTAAACTTAAGCTTATGGTTGCTGCAGGACTCGG	FRET
*ghrb*-cds-R	GAACCGTTGCCAGAGGATCCGGCGGTGCTTCCGGAGATAGAGCTGATTTATGGTG	FRET
*gh*-site1	GCUCAGUCCUGAAUCUCUUTT	RNAi site1
*gh*-site2	GGACAUGCACAAGGUGGAATT	RNAi site2
*gh*-site3	GCACAAGGUGGAAACAUAUTT	RNAi site3
*prl*-site1	CCGUACAUGUGUCACACCUTT	RNAi site1
*prl*-site2	GACGGGAUUCCCACAAGAUTT	RNAi site2
*prl*-site3	GAUCGACAGCUUCCUGAAATT	RNAi site3
*sl*-site1	CCAUCCAAGAUGCCAGUAATT	RNAi site1
*sl*-site2	GGAUCGAGCCUCUGAUCUATT	RNAi site2
*sl*-site3	GCACCAGACCUGUUGGAAUTT	RNAi site3

## Data Availability

The original contributions presented in this study are included in the article. Further inquiries can be directed to the corresponding author.

## Abbreviations

The following abbreviations are used in this manuscript:

*gh*	growth hormone
*prl*	prolactin
*prl2*	prolactin 2
*sl*	somatolactin
*ghr*	growth hormone receptor
*slr*	somatolactin receptor
*prlr*	prolactin receptor
*vip*	vasoactive intestinal peptide
*pomc*	pro-opiomelanocortin
*pou1f1*	pou Class 1 Homeobox 1
FRET	The Fluorescence Resonance Energy Transfer

## Appendix A

**Table A1:** Accession numbers of *gh*/*prl*/*sl* family and their receptors used in this study.

Species	Protein	Accession Number
*Cynoglossus semilaevis*	gh1	NP_001281140.1
	prl	XP_008328937.1
	prl2	XP_024913906.1
	prl2-like	XP_024912122.1
	smtla	XP_008306726.1
	ghra	NP_001281126.1
	ghrb	NP_001315166.1
	prlr	XP_008324293.1
	prlrb	XP_008334066.1
	prlr-like	XP_024918466.1
*Sparus aurata*	gh1	XP_030262341.1
	ghra	XP_030273854.1
	ghrb	XP_030292100.1
	prlrb	XP_030272992.1
	prlra	XP_030292731.1
*Homo sapiens*	gh1	NP_000506.2
	prl	NP_000939.1
	ghr	NP_000154.1
	prlr	NP_000940.1
*Mus musculus*	gh1	NP_032143.1
	prl	NP_001157002.1
	ghr	NP_001041643.1
	prlr	NP_001240710.1
*Danio rerio*	gh1	NP_001018328.2
	prl	NP_852102.2
	smtla	NP_001032795.1
	smtlb	NP_001032763.1_
	ghra	NP_001077047.1
	ghrb	NP_001104551.1
	prlra	XP_021324476.1
*Scophthalmus maximus*	prl	XP_035470397.1
	prl2	XP_035483075.1
	smtla	XP_035479871.1
	ghra	XP_035496335.1
	ghrb	XP_047185503.1
	prlra	XP_035464595.2
	prlrb	XP_035497061.1
*Oryzias latipes*	smtla	NP_001098260.1_
	prlra	XP_011479970.1
	prlrb	XP_004072141.1
	ghra	NP_001098560.1
	ghrb	NP_001116377.1
*Salmao salar*	prl	XP_008328937.1
	prl2a	XP_045578518.1
	slr	NP_001135089.1
*Oncorhynchus mykiss*	gh2	NP_001118162.2
	gh1	NP_001118161.1
	prl	NP_001118205.1
*Thunnus maccoyii*	gh1	XP_042247785.1
	prl2	XP_042259945.1
	smtla	XP_042270649.1
*Oreochromis niloticus*	prl	NP_001266715.1
*Poecilia latipinna*	prl	XP_014877782.1
*Takifugu rubripe*	smtla	XP_029700280.1
	prl	NP_001072092.1
	prl2a	XP_029681130.1
	prl2b	XP_029681130.1
*Scophthalmus maximus*	prl	XP_035470397.1
	prl2	XP_035483075.1
	smtla	XP_035479871.1
*Hippoglossus stenolepis*	prl-like	XP_047195737.1
*Oncorhynchus masou*	slr	NP_001281140.1
*Solea senegalensis*	prlra	XP_008328937.1
*Hippoglossus hippoglossus*	prlra	XP_034458243.1
	prlrb	XP_034450669.1
	prlr-like	XP_034466717.1
*Paralichthys olivaceus*	prlra	NP_001281126.1
	prlrb	NP_001315166.1
	prlr-like	XP_008324293.1
